# Integrative System Biology Analysis of Transcriptomic Responses to Drought Stress in Soybean (*Glycine max* L.)

**DOI:** 10.3390/genes13101732

**Published:** 2022-09-26

**Authors:** Amir Ghaffar Shahriari, Zahra Soltani, Aminallah Tahmasebi, Péter Poczai

**Affiliations:** 1 Department of Agriculture and Natural Resources, Higher Education Center of Eghlid, Eghlid 7381943885, Iran; 2 Institute of Biotechnology, Shiraz University, Shiraz 7144113131, Iran; 3 Department of Agriculture, Minab Higher Education Center, University of Hormozgan, Bandar Abbas 7916193145, Iran; 4 Plant Protection Research Group, University of Hormozgan, Bandar Abbas 7916193145, Iran; 5 Finnish Museum of Natural History, University of Helsinki, P.O. Box 7, FI-00014 Helsinki, Finland; 6 Faculty of Biological and Environmental Sciences, University of Helsinki, P.O. Box 65, FI-00065 Helsinki, Finland; 7 Institute of Advanced Studies Kőszeg (iASK), P.O. Box 4, H-9731 Kőszeg, Hungary

**Keywords:** co-expression analysis, drought stress, *Glycine max*, meta-analysis, transcriptome

## Abstract

Drought is a major abiotic stressor that causes yield losses and limits the growing area for most crops. Soybeans are an important legume crop that is sensitive to water-deficit conditions and suffers heavy yield losses from drought stress. To improve drought-tolerant soybean cultivars through breeding, it is necessary to understand the mechanisms of drought tolerance in soybeans. In this study, we applied several transcriptome datasets obtained from soybean plants under drought stress in comparison to those grown under normal conditions to identify novel drought-responsive genes and their underlying molecular mechanisms. We found 2168 significant up/downregulated differentially expressed genes (DEGs) and 8 core modules using gene co-expression analysis to predict their biological roles in drought tolerance. Gene Ontology and KEGG analyses revealed key biological processes and metabolic pathways involved in drought tolerance, such as photosynthesis, glyceraldehyde-3-phosphate dehydrogenase and cytokinin dehydrogenase activity, and regulation of systemic acquired resistance. Genome-wide analysis of plants’ cis-acting regulatory elements (CREs) and transcription factors (TFs) was performed for all of the identified DEG promoters in soybeans. Furthermore, the PPI network analysis revealed significant hub genes and the main transcription factors regulating the expression of drought-responsive genes in each module. Among the four modules associated with responses to drought stress, the results indicated that GLYMA_04G209700, GLYMA_02G204700, GLYMA_06G030500, GLYMA_01G215400, and GLYMA_09G225400 have high degrees of interconnection and, thus, could be considered as potential candidates for improving drought tolerance in soybeans. Taken together, these findings could lead to a better understanding of the mechanisms underlying drought responses in soybeans, which may useful for engineering drought tolerance in plants.

## 1. Introduction

Drought stress is one of the main environmental stress conditions limiting crop production and plant distribution throughout the world. Soybeans (*Glycine max* L.), which constitute a major source of proteins, unsaturated fats, carbohydrates, and fibers, are one of the most significant legume crops and are capable of providing nutritional security for the global population, as well as being important for biotechnological applications [1]. The timing, duration, and severity of drought stress are important factors in soybean yield and quality. When plants are constantly exposed to drought stress, they can suffer from several impairments, such as oxidative injury, membrane system damage, cellular ion leakage, and protein denaturation [2]. Previous studies have shown that the photosynthesis rate and CO_2_ uptake of a plant decline during drought stress, adversely affecting biomass accumulation and yield [3,4]. Therefore, plants have evolved complex molecular, physiological, and biochemical strategies to adapt to the effects of drought stress [5,6]. In this regard, water stress causes the production of reactive oxygen species (ROS), and excessive ROS lead to oxidative stress, which inhibits plant growth and, ultimately, causes cell death [7]. An effective and direct approach to enduring drought stress is to reduce water loss by closing stomata. Plants’ responses to drought stress are modulated by multiple factors, including osmotic regulation, environmental signals, photosynthesis, hormone regulation, CO_2_ concentration, and respiration [8]. Drought resistance is a complex trait controlled by several different genes. Despite extensive research on drought resistance in plants, it is still not fully understood and needs more investigation [9]. The genes differentially expressed during drought stress include those encoding critical enzymes for the biosynthesis of hormones, proteins involved in osmotic adjustment and cell protection, and various cell-signaling proteins such as kinases, phosphatases, and transcription factors [10]. 

Omics is a multidisciplinary field of study that is largely performed through the application of several high-throughput technologies, which mainly involve qualitative and/or quantitative detection of novel or previously identified genes, transcripts, transcription factors, proteins, metabolites, and other parameters [11,12]. With the advent of high-throughput sequencing technologies, large-scale genomic data are being generated and deposited in public domain databases by various research organizations around the world [13,14]. Most of these datasets are related to the expression of genes from various experiments conducted to understand the complex biological mechanisms of plants under biotic and abiotic stresses, including widespread transcriptional and metabolic events. Physiological and molecular studies of stresses suggest a common network of multiple signaling pathways in plants [15]. Therefore, progress in new techniques of functional genomics—such as microarrays and RNA-Seq technologies—allows the analysis of gene regulation and model species under various stresses. The RNA-Seq and microarray approaches are fundamentally different from one another in terms of gene expression measurements. The former allows for direct sequencing of the whole transcriptome, while the latter only processes predefined transcripts/genes through hybridization [16]. RNA-Seq is superior in detecting low-abundance transcripts, differentiating biologically critical isoforms, and allowing the identification of genetic variants. Moreover, this approach does not depend on genome annotation for prior probe set selection, and it avoids the related biases introduced during hybridization of microarrays, such as background noise [17]. Despite the benefits of RNA-Seq, microarrays are still the more common choice of researchers to perform transcriptional profiling experiments. RNA-Seq technology is new to most researchers. This method is also more expensive than microarrays, and its data storage is more challenging. In addition, there is a lack of optimized and standardized protocols for its analysis, in spite of the availability of multiple computational tools [16]. Thus, microarrays have become the predominant platform for transcriptome profiling, because of their relatively high sensitivity, specificity, ease of analysis, accuracy, throughput, and cost-effectiveness [18]. DNA microarrays are helpful in studying complex ecological interactions; hence, we used this method to conduct the comprehensive transcriptome analysis of drought-tolerant soybeans in our research. [19]. In recent years, omics data and progressive statistical analyses such as meta-analyses and computational systems biology have created excellent opportunities to conquer biological complexity [20,21]. For instance, meta-analysis is a potent strategy that integrates transcriptomic data to recognize core gene sets and regulate their complex traits [22]. Therefore, researchers are able to obtain more reliable results by integrating information from multiple sources. Furthermore, they can investigate the expression of thousands of genes and their co-expression partners under diverse abiotic stresses [23]. Microarray data are frequently used to elucidate gene expression profiles and detect modules in genetic network analysis, which allows the simultaneous analysis of a many genes and samples. Moreover, genetic network analysis provides a way to discover metabolic and gene expression patterns at the genomic scale, as well as the possibility of analyzing the expression of unknown genes compared to sequences in databases [24].

Analysis of gene co-expression networks must be carried out to understand the interrelationships between the selected DEGs and identify genes with similar expression patterns that may participate in specific biological functions [25]. Weighted gene co-expression network analysis (WGCNA) is a powerful approach for exploring transcriptomic data and deciphering co-expression patterns among genes. The WGCNA technique deals primarily with the identification of gene modules by using the gene expression levels that are highly correlated across samples [26]. This approach has been successfully utilized to understand transcriptional regulation in many plant species, such as maize (*Zea mays* L.), rice (*Oryza sativa* L.), tomatoes (*Solanum lycopersicum* L.), *Arabidopsis thaliana*, and soybeans [27,28,29,30,31,32,33]. Co-expression networks are scale-free networks of nodes representing genes that are connected by edges whenever they are significantly co-expressed [25,26]. In such a network, highly connected genes are called hub genes, which are expected to play a significant role in understanding the biological mechanisms of responses to biotic and abiotic stresses in plants [34,35]. Here, we should also emphasize that the use of co-expression network analysis is an effective technique to develop a hypothesis. The widely used proof-of-hypothesis approach is direct selection for yield stability based on an experimental system adapted to adverse environmental conditions. This system is a time-consuming, labor-intensive process and is more challenging for abiotic-stress-related traits due to its low heritability and the high influence of different environmental conditions [36]. Therefore, an alternative option is molecular breeding using co-expression analysis, which can identify functional pathways and then accelerate the development of tolerant cultivars.

System biology approaches use correlations between genes to cluster genes with similar expression profiles under multiple experimental conditions into co-expression modules. Gene co-expression modules reflect DEGs that contribute to the interrelated biological pathways and processes. Such gene modules may be conserved across species and even various abiotic stresses. In the present study, we performed a large-scale meta-analysis of stress-response studies using microarray gene expression data to detect DEGs involved in responses to drought stress. Although meta-analysis has proven to be useful in discovering differentially expressed genes (DEGs), co-expression network analysis is a critical step in the selection of informative genes and predicting gene functions. As a result, systems biology analysis was employed to identify hub genes and provide further insight into the mechanisms related to soybeans’ response to drought stresses. These results will be valuable resources for studying drought resistance in soybeans, as well as a basis for further research on genes involved in drought resistance. The aim of this study is the identification of core gene sets that regulate the drought tolerance between stress and normal conditions in soybeans.

## 2. Materials and Methods

### 2.1. Data Collection and Pre-Processing

The soybean microarray experimental datasets under drought stress were collected from Gene Expression Omnibus with the platform GPL13674 (http://www.ncbi.nlm.nih.gov/geo/query/acc.cgi?acc=GPL13674, [accessed on 3 June 2011]). This platform contains 128 experimental samples and 10 series records on 66,660 probesets generated using the Affymetrix Glycine max Gene 1.0 ST Array. A total of 30 vegetative tissue samples related to drought stress were collected for further study. The number of selected controls and treatments for each stress condition, along with the experimental sample IDs, is given in Table 1. The conditions, stages, and tissues are as follows:

GSE29663 and GSE40627: Seedlings of soybean plants (cultivar: Williams 82) were grown in pots containing Supermix. The pots were watered once per day under greenhouse conditions (continuous 30 °C temperature, photoperiod of 12 h/12 h). Soybean plants at the V6 stage (28 days after sowing, containing 7 trifoliate leaves) were withheld from watering to initiate the drought treatment and, finally, leaf samples were collected for RNA extraction. 

GSE65553: Soybean plants (cultivar: Williams 82 and DT2008) were separately grown in pots containing vermiculite under well-watered conditions in a controlled greenhouse (continuous 30 °C temperature, photoperiod of 12/12 h). When the plants reached the V2 stage (14 days after sowing, containing 2 trifoliate leaves), drought treatment was imposed by withholding water. After the dehydration treatment, plants dehydrated for 2 and 10 h were collected, and the roots were separated from the shoots.

Initially, raw CEL files of these selected microarray datasets were pre-processed using the Robust Multichip Average (RMA) algorithm available in the affy Bioconductor package of R [37,38]. The pre-processing stage included background correction, quantile normalization, and summarization using the median polish approach [39]. Finally, the log_2_ scale-transformed expression data from the RMA for these collected microarray samples were applied for further statistical analysis. Batch effects are one of the main sources of non-biological variation that affect the outcomes of meta-analyses [40,41]. We used the SVA package and empirical Bayes method in R software to correct the batch effect [42,43].

### 2.2. Screening and Identification of Differentially Expressed Genes

Gene expression data were filtered by removing genes with low transcript levels in all 30 vegetative tissue samples. A total of 11,205 genes were applied to identify the differentially expressed genes (DEGs), which were then analyzed using weighted gene co-expression network analysis (WGCNA). The metaDE R package and RankProd method were used to obtain the “base mean” value to identify DEGs [44]. The standard for screening was the up- and downregulated genes with a *p*-value ≤ 0.05, which were considered to be DEGs [45].

### 2.3. Weighted Gene Co-Expression Network Analysis

The co-expression network of all DEGs was constructed using a WGCNA R package (V 1.51) to further elucidate the functions and mechanisms of genes in soybeans under drought stress [25]. First, Pearson’s correlation coefficients were calculated for all pairwise genes, and a soft threshold was then obtained to construct a similarity matrix. Subsequently, the similarity matrix was converted to adjacency matrices by raising them to the power (β) that highly approximated the scale-free behavior of the resultant networks. Finally, the adjacency was transformed into a topological overlap matrix (TOM), and the genes were hierarchically clustered based on TOM similarity. To identify highly correlated modules and assign genes to them, a dissimilarity matrix was obtained (dissTOM) and used to represent the distances between genes [44]. The dynamic hybrid tree-cut algorithm was utilized to cut the hierarchal clustering tree and define modules as branches from the tree cutting [26]. Moreover, the module eigengene was applied to summarize the expression profile of each module. The modules were defined with a minimum module size of 30 genes and a merged cut height of 0.3 to avoid abnormal modules in the dendrogram. Additionally, the soft-thresholding power (β = 10) was chosen based on the lowest power for which the scale-free topology fit index reached a high value.

### 2.4. Identification of Hub Genes

Network visualization of each module was performed using the Cytoscape software (V. 3.6.1) [46] with a cutoff value of the weight parameter obtained from the WGCNA, set at ± 0.30. Hub genes are defined as central genes with high correlation in the candidate modules in each stage. The central genes were identified using the cytoHubba plugin of Cytoscape by visualizing the 30 nodes with the highest interaction in the biological network [47]. Here, the calculation algorithms of maximal clique centrality (MCC) were used as the most effective methods [48].

### 2.5. GO and Pathway Functional Enrichment Analyses

To identify the enriched biological processes and metabolic pathways involved in drought tolerance, we routinely performed Gene Ontology (GO) and Kyoto Encyclopedia of Genes and Genomes (KEGG) enrichment analysis. Gene Ontology analysis included three categories: molecular function (MF), biological process (BP), and cellular component (CC). The clusterProfiler R package was utilized to perform GO and KEGG analyses. The results showed statistical significance at a *p*-value ≤ 0.05 and count > 2. Finally, the results were exhibited using the “heat map” R package.

### 2.6. Cis-Acting Element Analysis

In order to obtain the promoter regions and the genomic sequences, 1500 bp from the upstream flanking regions of shared DEGs were extracted from Ensembl Plants BioMart version 54 (last updated on July 2022) (http://plants.ensembl.org). Briefly, all upstream sequences of each gene to the beginning of the contig sequence were considered to be promoter sequences. Then, the MEME version 5.4.1 (25 August 2021) (meme.nbcr.net/meme/intro.html) [49] database was used to identify the conserved motifs on the sequences using its default parameters, except for the maximum number of motifs (11). In the next step, we used the Tomtom v 5.0.1 tool (http://meme-suite.org/tools/tomtom) to eliminate redundant motifs and determine known CREs based on the motif database of JASPAR CORE 2022 Plants [50], with threshold E-value cutoff of 0.05. Finally, the GMO tool (http://meme-suite.org/tools/gomo) was also applied to determine the biological roles of the drought-resistant motifs [51].

### 2.7. Identification of Transcription Factor Families

Selected genes were further analyzed and described using WGCNA. To identify transcription factor families involved in soybeans’ drought tolerance, sequences of DEGs were obtained by BLASTx search and then examined against the iTAK database (http://bioinfo.bti.cornell.edu/cgi-bin/itak/index.cgi) [44].

### 2.8. Protein–Protein Interaction (PPI) Networks 

The network analysis of protein–protein interactions (PPIs) was performed to uncover plausible interactions among proteins with candidate hub genes involved in the drought-tolerance pathways. The STRING version 11.5 (12 August 2021) (Search Tool for the Retrieval of Interacting Genes/Proteins, http://string-db.org/) interactome database with default parameters (lowest required interaction score = 0.150) was employed to enable PPI network analysis. Low confidence was applied to simplify the network and to study significant and key connectivities. Finally, the Cytoscape software was used to visualize the interaction networks [52].

### 2.9. Validation Analysis

To validate the results of the meta-analysis, a leave-one-out cross-validation (LOOCV) was implemented on the expression values of hub genes derived via co-expression analysis. In the cross-validation process, an initial dataset was split into a training set and a test set. Then, one sample from the initial dataset was consecutively discarded for test, and the others for training [53,54].

## 3. Results 

### 3.1. Pre-Processing and Identification of the Drought-Responsive Core DEGs

**Figure 1:**
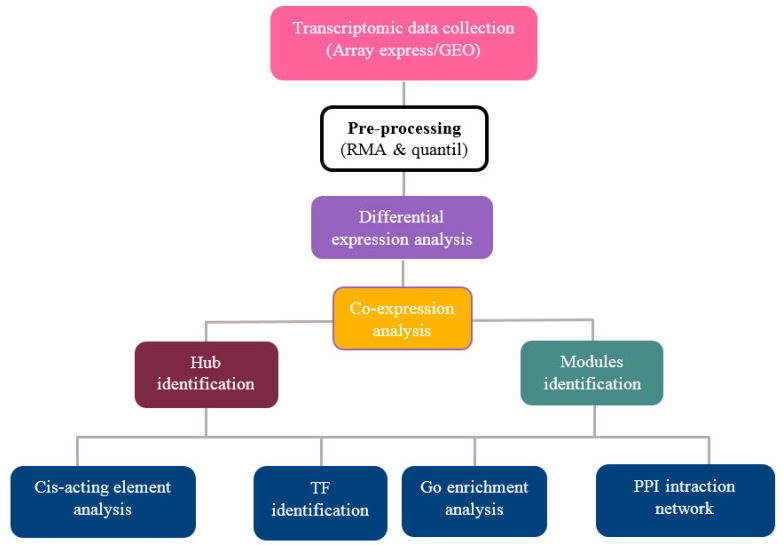
Workflow for data collection, pre-processing, and co-expression network analysis to assess aspects of the responses of soybeans to the effects of drought stress.

In this study, we analyzed the gene expression profiles of soybeans for drought stress response using different datasets. A schematic workflow of the analysis is described in Figure 1, from data collection and processing, to differentially expressed gene prediction, potential module detection, hub characterization, and network construction. 

**Figure 2:**
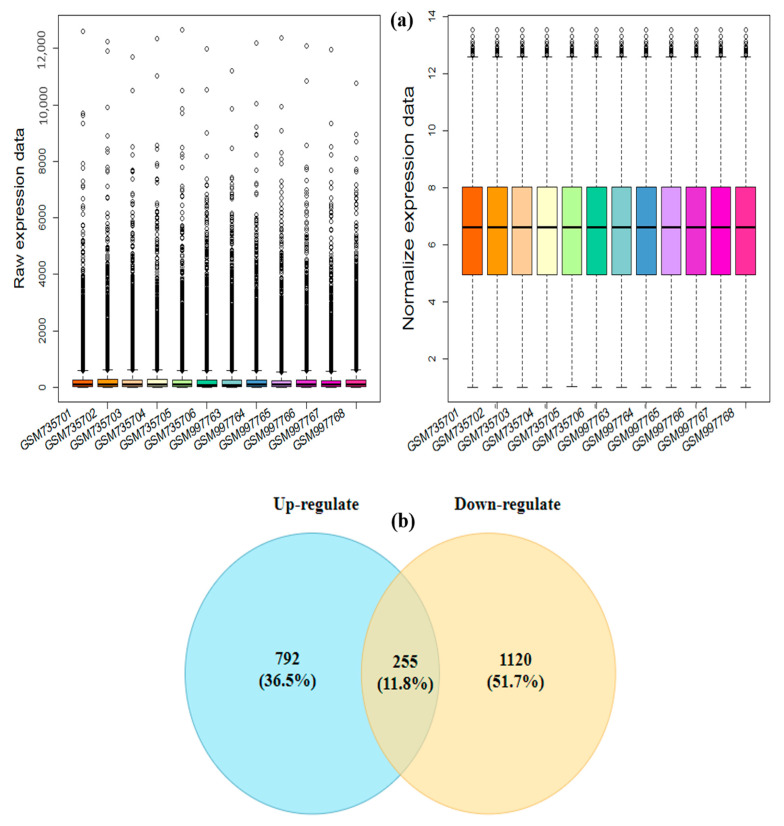
(**a**) Pre-processing of microarray studies. The E-GEOD-40627 study boxplot related to drought stresses in the Affymetrix platform, with 6 control samples and 6 treatment samples, was drawn for the pre- and post-normalization stages. The boxplot is presented after normalization, where all comparisons that are not significant or are not equal to the change threshold are converted to a log_2_ value to remove possible errors. The black lines of the boxplot are almost on the same straight line, indicating a high level of normalization. The horizontal axis stands represents the control and different treatment samples, while the vertical axis represents the expression values. The black line in the box represents the median expression for each sample. (**b**) Venn diagrams illustrating the numbers of down- and upregulated DEGs in the drought stress studies. The intersection in grey represents the DEGs common between the two datasets.

The raw data related to drought stress were processed using intra-study RMA and quantile normalization. The relative quality of different samples within the dataset was examined using the Affymetrix Bioconductor package in R. The samples were split into control and stress conditions in each study to identify DEGs. To remove heterogeneity between studies, batch effect correction was performed on gene expression data, followed by batch variation between studies of reduction, and actual gene expression values were estimated (Figure 2a). Finally, normalized datasets were used to detect differentially expressed genes under drought stress using the RankProd method. A total of 2168 differentially expressed genes were identified among the different samples in the datasets (adjusted *p*-value < 0.05). Among them, 864 differentially expressed genes were significantly upregulated, whereas 1303 were downregulated across the datasets (Table S1). The Venn diagrams in Figure 2b show the numbers of specific and commonly regulated genes between the drought stress and control conditions. 

### 3.2. Co-Expression Analysis and Module Identification under Drought Stress

**Figure 3:**
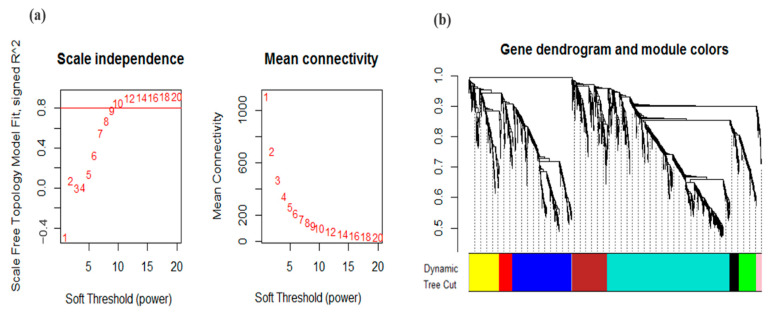
(**a**) Determination of soft-thresholding power (β) in the weighted gene co-expression network analysis (WGCNA) and module identification. The left panel shows the analysis of the scale-free fit index for various soft-thresholding powers (β). The right panel displays the analysis of the mean connectivity (degree, y-axis) for various soft-thresholding (x-axis) powers. (**b**) Weighted gene co-expression network analysis by the dynamic tree-cutting method. Dendrogram of all DEGs clustered based on a dissimilarity measure (dissTOM). The branches correspond to modules of highly interconnected groups of genes. Each color represents one specific co-expression module, and the tips of the branches represent genes.

The principal purpose of co-expression network analysis is to identify clusters or modules of densely interconnected genes that can be analyzed by searching for patterns in connection strength [55]. In the present study, to identify the expression of genes related to drought stress in soybeans, a gene co-expression network was constructed using the WGCNA R package. Weighted gene co-expression networks do not depend on a hard threshold, because using a hard threshold would be likely to lead to loss of information and sensitivity [26]. Therefore, soft-thresholding power with a scale-free model fitting index *R*^2^ < 0.8 (Figure 3a) was applied to maximize the scale-free topology. The DEGs based on the dynamic tree-cutting algorithm were grouped into eight modules involved in response to drought stress, ranging in size from 36 to 911 genes per module (Figure 3b and Table 2).

**Figure 4:**
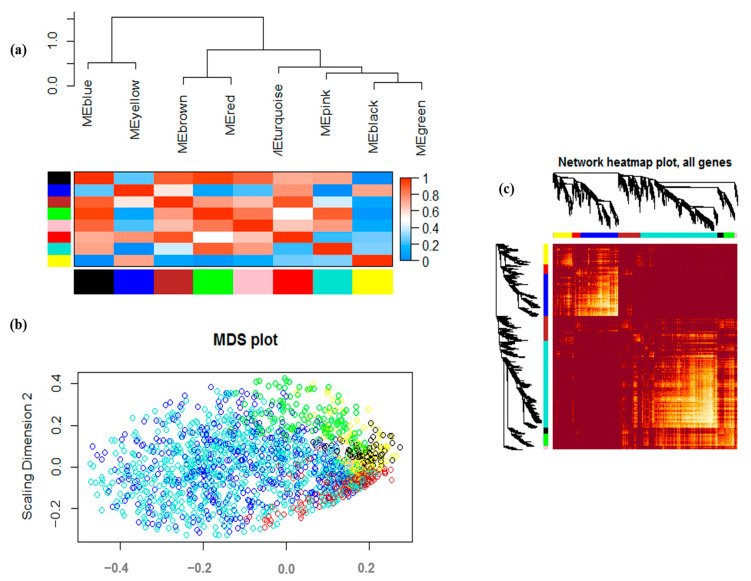
(**a**) The module eigengene adjacency shown by hierarchical clustering and heatmap. A module eigengene (ME) summarizes the gene expression profile of each module. In the heatmap of module–module relationships, the progressively more saturated blue and red colors indicate high co-expression interconnectedness. (**b**) Multidimensional expansion map (MDS) of each gene located in a module of the soybean drought stress dataset. MDS plot demonstrating the similarity of gene expression patterns between different modules. (**c**) In the topological overlap matrix (TOM) plot, darker red color represents low overlap, while progressively lighter color represents higher overlap among DEGs. Blocks of darker colors along the diagonal correspond to the modules. The gene dendrogram and module assignment are also shown along the left-hand side and the top.

These eight modules yielded two main clusters: one contained two modules, while the other contained the other six modules, which can also be divided into four sub-clusters. This result was also supported by the heatmap plot of the adjacencies (Figure 4a). Genes in green, pink, and brown modules showed similar expression patterns. In the eigengene adjacency heat map, the slope of the variance in color from black to yellow represents the connectedness of genes for various modules, from strong to weak. As is evident from the corresponding red highlights in the drought stress heatmap in Figure 4b, the brown and blue modules exhibited the strongest gene–gene interconnectedness based on the TOM dissimilarity distances. According to the multidimensional scaling (MDS) in the soybean drought stress data (Figure 4b), the genes in most modules—including the blue, turquoise, brown, green, and pink modules—showed similar expression patterns. Moreover, based on the topological overlap matrix (TOM) shown in Figure 4, dark colors represent low overlap, while progressively light and yellow colors represent higher overlap. Blocks of light colors along the diagonal are the modules (Figure 4c). 

The enrichment analysis for the biological process categories of genes comprising these modules was performed to investigate how plants respond to drought stress (Table S2). Several significant terms with *p*-values < 0.05 were identified. The turquoise, brown, and blue modules formed the largest cluster of genes enriched in functions related to responses to water-deprivation biological processes. Upregulated DEGs included *NNRP-B* and *GmMAPK3-2*, while downregulated DEGs included *PIP1-6* and *HIS1-3*; thus, genes in these modules specifically regulate drought tolerance in soybeans. We observed that in all of the detected modules, the gene expression levels were higher under drought stress than under control conditions, including glutamine synthetase (*GLYMA05G37760.1*), asparagine synthetase (*GLYMA11G27480.1*), galactolipids transferase (*GLYMA01G32750.1*), thiamine thiazole synthase (*GLYMA10G39740.1*), ATP synthase, and functionally unknown proteins. These results indicate that amino acid metabolism, fatty acid biosynthesis, and energy supply are closely related. In addition, the gene *hcf* encoding photosystem II and photosystem I in photosynthesis was linked to the turquoise, red, and black modules containing ferredoxin (*GLYMA_12G1694001*) and glyceraldehyde-3-phosphate dehydrogenase (*GLYMA02G36370.1*), which play key roles in respiration. These modules probably have a high positive correlation with leaves (r = 0.8; *p* < 0.05), because photosynthesis is responsible for CO_2_ fixation in triose phosphate, which feeds sucrose synthesis [56,57]. Hence, our findings support a strong transcriptional co-regulation of sucrose synthesis and photosynthesis genes, suggesting that these modules are involved in photosynthesis, respiration, and energy metabolism. Finally, many genes detected in previous studies—such as genes involved in protein folding (*Rotamase CYP4*), regulation of lignin biosynthesis (*MYB, XIP1*), transcription factors (*AP2-EREBP, LHY2b*), silencing proteins (*EMB2777*), abscisic acid signaling pathways (*TCH2, PIP, ACO3*), auxin (*Aux/IAA*), peroxidase precursors (*PRX2*B), and some proteins with unknown functions (GLYMA03G024400, GLYMA08G150400, GLYMA11G099700, and GLYMA11G215500) that cause responses to environmental stresses,—were assigned to this module. Lignin in plant biomass is the main contributor to cell-wall recalcitrance; thus, low lignin can substantially improve the scarification efficiency of plant cell walls, playing critical roles in plant growth, defense, and morphology [58,59]. Taken together, the results of previous studies indicate that engineered plants with low lignin contents confer adaptation to drought tolerance via an ABA-dependent pathway [60].

### 3.3. Identification of Hub Genes and Enrichment Analysis

**Figure 5:**
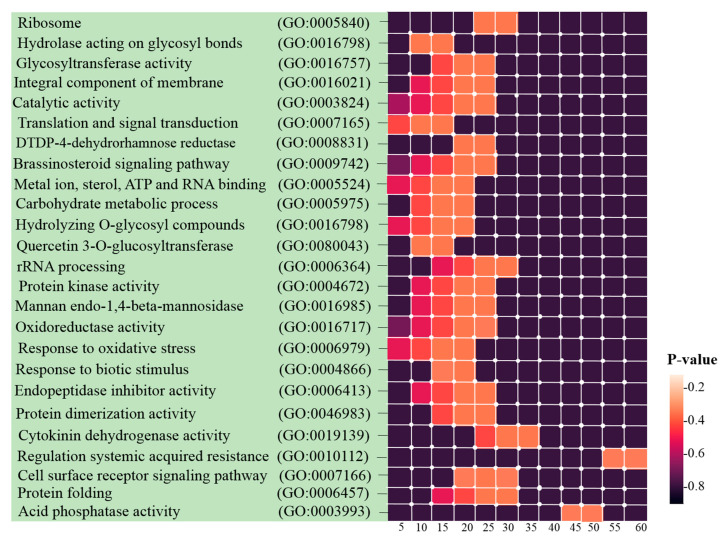
Binder and heatmap plots for the Gene Ontology analysis. GO enrichment analysis of hub genes was retrieved using DAVID. The most significantly (*p* ≤ 0.05) enriched GO terms in biological process (BP) branches related to defense responses to drought are presented. The horizontal axis shows the number of genes based on the *p*-value, while the vertical axis represents the GO terms.

The network of co-expressed modules was constructed to identify the genes with high connectivity (known as hub genes) and genes with central roles in the network. Owing to their central location within the network clusters, the hub genes were considered to be critical components of the networks. To investigate the relationships between the enriched modules and drought stress, the hub genes were identified by ranking the connectivity of each node to each module, and then they were further validated by their degree of overall intramodular centrality (a high value confirmed hub status). The top 20 hub genes were chosen for each module, and significant enrichment was performed for 160 hub genes (Table S3). As mentioned in the previous section, these hub genes were strongly enriched in translation, photosynthesis, protein folding, hydrolase activity, and integral components of membrane activity, cytokinin dehydrogenase activity, protein kinase activity, oxidoreductase activity, regulation of systemic acquired resistance, and oxidoreductase activity, resulting in the reduction of molecular oxygen to two molecules of water (Figure 5). 

Among the four gene clusters associated with responses to drought stress, the results indicated that *GLYMA_04G209700* in the turquoise module, *GLYMA_02G204700* in the green module, *GLYMA_06G030500* in the pink module, and *GLYMA_01G215400* and *GLYMA_09G225400* in the red module had high degrees of interconnection. However, some hub genes—including *GLYMA02G215700*, *GLYMA01G124500*, *GLYMA03G181700*, *GLYMA01G232400*, *GLYMA01G142400*, and *GLYMA02G203300*—were of unknown function; thus, they may be considered as potential candidates for further studies.

### 3.4. Identification of Transcription Factors

**Figure 6:**
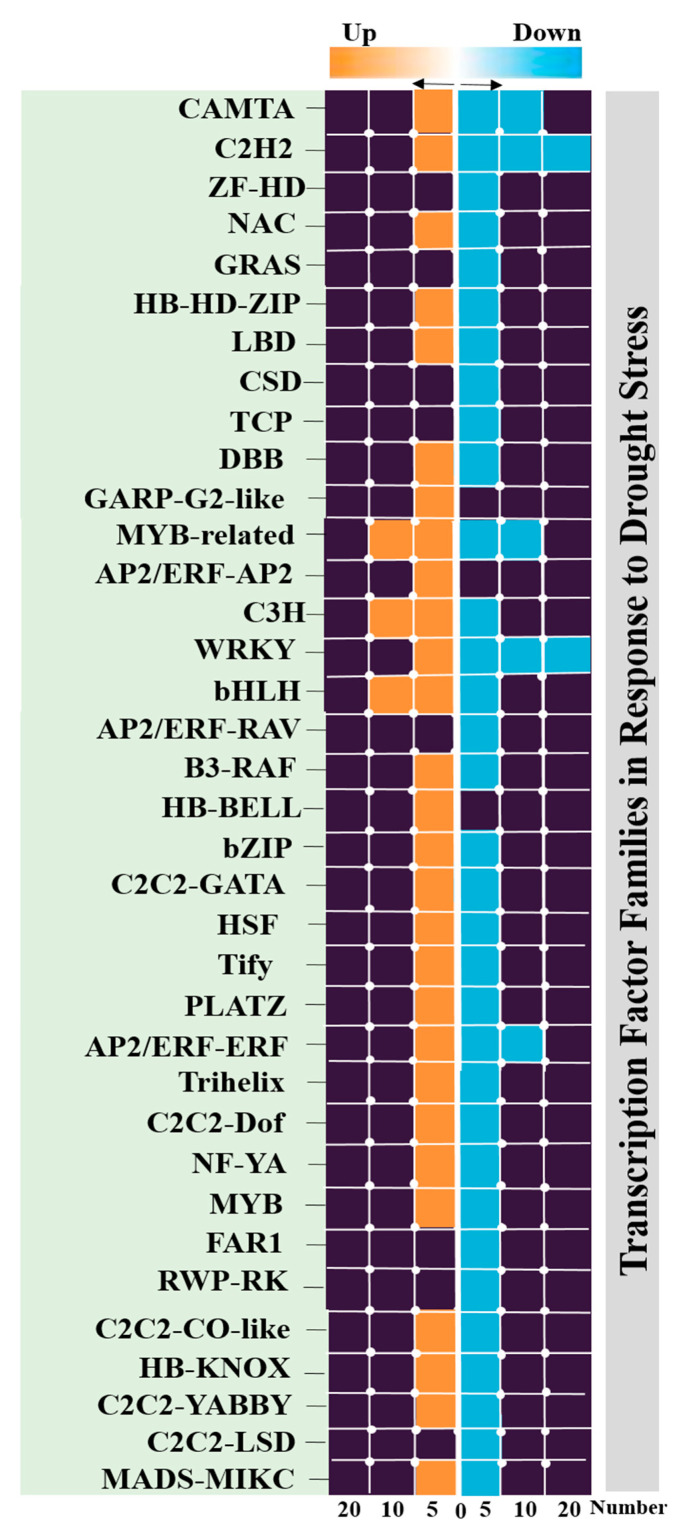
Bar plot showing the distribution of TF families identified in DEGs. The numbers of up- and downregulated genes in TF families. The y-axis refers to the number of genes, while the x-axis shows TF families.

Transcription factors are essential players in biotic and abiotic stresses through transcriptional regulation. Therefore, to acquire a better understanding of the potential impact of transcription factors in the control of drought-tolerance genes and their contribution to the manipulation of complex metabolic pathways, the sequences of genes that are differentially expressed in a given pathway were used to predict the possible binding sites using the iTAK database. A total of 174 TF genes for drought stresses were found, all of which belonged to 36 TF families that are directly or indirectly involved in signaling and response to stresses (Table S4). Members of the *MYB*, *bHLH*, *C2H2*, *MYB-related*, and *AP2/ERF-ERFn* families were the top classes (Figure 6). 

Among these transcription factor families, only 20 families (65 DEGs) were significantly upregulated, including orphan, *MYB*, *AP2/ERF-ERF,* and *WRKY*. In addition, a total of 21 TF families (109 DEGs) were significantly downregulated, including *bHLH*, *C2C2-MYB-related,* and *C2C2-Dof*. Moreover, these transcription factor families were shown to be involved in positive regulation of transcription and DNA templates. Since TFs have been well-characterized as involved in abiotic stress tolerance, we further extended the study to detect significant hub genes and TFs in each module. We found that the transcription factor abundance was higher in the turquoise module (86 TFs) compared to the blue module (36 TFs), yellow module (17 TFs), and brown module (14 TFs). As mentioned above, we selected only the top 20% of gene modules with high degrees of connectivity. A total of 160 DEGs were identified as hub genes (Table S3), including 11 TFs representing distinct families, such as WRKY, MYB-related, HB-BELL, MYB, C2C2-Dof, C3H, bZIP, DBB, and bHLH TFs. These families of crucial TFs are involved in ABA signaling pathway and stomatal closure [61,62]. This indicates the role of the turquoise, blue, yellow, and brown modules in water stress management by regulating stomatal closure and ABA signaling pathways to reduce water loss, thereby minimizing photosynthesis activity and shifting to other metabolic pathways to meet energy demands.

### 3.5. Cis-Acting Elements Analysis and Motif Identification

We performed promoter motif analysis to identify potential regulatory elements associated with the drought stress resistance pathway. Initially, the 1500 bp upstream flanking regions of the DEGs were analyzed to find conserved motifs and consensus cis-acting regulatory elements. Then, 11 significant motifs with lengths ranging from 11 to 50 nt were identified in the promoters of DEGs using MEME (Table 3). The GOMO analysis for the motifs found by MEME detected various biological functions (Table S6). As shown in Table 3, Gene Ontology analysis indicated that these motifs are involved in responses to water deprivation, amino acid phosphorylation, regulation of transcription, photosynthetic electron transport in photosystem I, oligopeptide transport, the initiation of DNA replication, and developmental growth. Based on the results, it seemed that the C2H2, Dof, BBR/BBC, and MYB transcription factor families were the most significant transcription factors, because 85% of the differentially expressed genes had a binding site in these promoters (Table S5). This analysis also highlighted motifs related to the drought tolerance signaling pathway. Moreover, these motifs were involved in molecular functions including transcription factor activity, protein serine/threonine kinase activity, protein binding, and protein heterodimerization activity (Table 3). After filtering (*p*-value ≤ 0.05), many motifs were found in promoters related to C2H2 zinc finger factors (*DOF5.8*), the most notable of which were the motifs MA1281.1 and MA1278.1 in response to drought stress DEGs.

### 3.6. Protein–Protein Interactions and Selection of Key Genes

**Figure 7:**
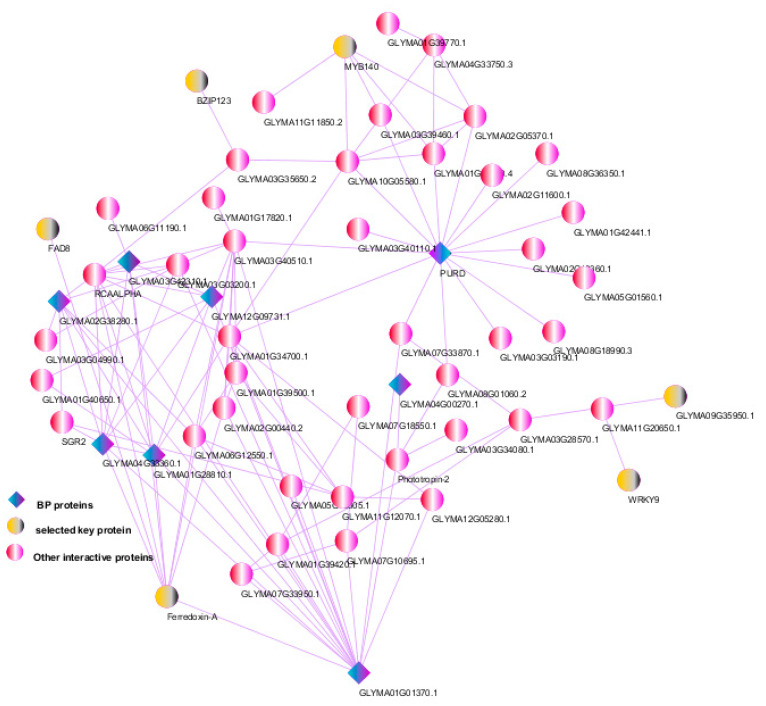
A protein–protein interaction network highlighting the hub genes involved in drought stresses in soybeans. The most significant hubs are ranked based on their importance in the network. The yellow nodes represent the selected key genes, while the turquoise nodes represent the significant biological functions in the network.

Understanding the regulatory gene network that is responsive to water stress can help both researchers and breeders in manipulating plants to improve stress resistance and productivity. The protein–protein interaction (PPI) network analysis comprised 80 drought-related hub genes. Minimum default settings were used to reduce the number of interacting proteins and the complexity of the network (Figure 7). The network showed 79 nodes and 93 edges during drought stress. Some key genes with a high number of interactions (>15)—including *PURD*, *RCAALPHA,* and *GLYMA01G01370.1*—were detected. In addition, some genes that play an important role in biological pathways—including metabolic pathways, photosynthesis, protein–chromophore linkage, reductive pentose–phosphate cycle, photosystem I, phosphoribulokinase activity, oxidoreductase activity, and response to light stimulus—were detected, such as GLYMA03G42310.1 (Gma.25294), GLYMA01G28810.1 (Gma.55002), GLYMA04G33360.1 (Gma.18151), GLYMA12G29090.2 (Gma.25330), GLYMA02G38280.1 (Gma.62172), GLYMA01G01370.1 (Gma.31315), GLYMA12G09731.1 (Gma.55256), and GLYMA08G41570.1 (Gma.1578). The results of the network analysis also indicated the connections of some TFs with other molecules. Some of the key upregulated hub (i.e., highly interacting) proteins that may play important roles in drought stress response included *WRKY9* (GLYMA01G06550.1), *MYB140* (GLYMA09G29800.3), *FAD8* (GLYMA03G07570.1), *Ferredoxin-A* (GLYMA03G07570.1), and GLYMA09G35950.1 (*GmCKX6-1*). The yellow nodes indicate the selected hub genes in the network. Other interactive proteins (pink nodes) at the protein–protein level were commonly regulated with the abiotic stresses and may be considered to represent general plant stress states. 

### 3.7. Leave-One-Out Cross-Validation of Hub Genes

The LOOCV was applied to validate the results of the meta-analysis and evaluate whether or not the hub genes could be used to distinguish between samples under drought stress and control conditions. The results indicated that the control and stress samples were correctly classified based on the expression levels of the top-ranked genes, and the classification accuracy was 93.08% with an area under the curve (AUC) of 0.917 (Figure S1).

## 4. Discussion

Drought stress greatly affects grain production during the transition from vegetative to reproductive development in the majority of crops—especially soybeans [63,64]. Thus, a complete understanding of the physiological, biochemical, and gene-regulatory networks associated with water-deficit stress tolerance at these different stages of vegetative growth in soybeans is essential for breeding drought-tolerant cultivars. However, the complex adaptive mechanisms underpinning water-deficit stress tolerance from vegetative growth to the reproductive development stage have remained elusive, despite recent advances in molecular biology approaches [65]. Improving the drought tolerance of soybeans is very significant, and more research is needed to explore and understand drought stress. In this study, we employed a microarray-based approach to perform a comprehensive transcriptomic analysis of drought-tolerant datasets, which were used at the vegetative growth stages to identify key regulatory genes and gene co-expression networks involved in soybeans’ drought stress responses. Recently, the availability of several bioinformatics approaches and statistical tools has helped researchers to identify key biological processes and metabolic pathways involved in tolerance to biotic or abiotic stresses. Our findings not only provide informative clues for the elucidation of drought stress tolerance in soybeans, but also represent a valuable resource and basis for the identification of candidate drought-resistance genes.

The present investigation was carried out with the aim of understanding the key players in drought tolerance in soybeans, using efficient co-expression network analysis approaches. The datasets contain gene expression profiles from microarray data of *Glycine max* under control and drought stress conditions. WGCNA is a powerful R package that divides the core DEGs in different modules based on correlation between co-expressed genes involved in specific metabolic pathways [25]. In the present study, we first identified drought-responsive core DEGs by cross-comparison of various transcriptome datasets of soybeans under water-deficit stress conditions (Figure 2b). Given that the expression of many DEGs was affected by drought treatment, WGCNA was used to construct a gene co-expression network to mine the main genes and reveal the key modules involved in soybeans’ responses to drought stress in the vegetative stages. WGCNA of these core DEGs was divided into eight modules, each of them contributing to drought tolerance via a unique metabolic pathway. We observed that all of the detected modules—especially the brown, blue, and turquoise modules—had positive correlations with drought stress treatment; thus, genes in these modules positively regulate drought tolerance in soybeans (Figure 3). The functions of DEGs with known biological functions could be predicted according to their module, and this analysis found a series of biological processes that were affected by water-deficit stress conditions. To obtain deeper insights into how these modules participate in drought tolerance, we performed GO and KEGG analysis of each module separately. 

By comparing our results with those of previous studies, similar biological processes were detected in plants’ responses to water stress. It is well known that partial closure of the stomata with sufficient CO_2_ input maintains photosynthesis and significantly reduces drought stress under all conditions evaluated. The probable explanation for this is that drought stress damages the photosynthetic organs and alters vegetative structures, thereby reducing the photosynthetic activities of plants and adversely affecting their growth [66]. Moreover, stomatal closure restricts the entry of CO_2_ and causes physiological damage during drought stress by facilitating ribulose 1,5-bisphosphate regeneration and adenosine triphosphate (ATP) production in photosynthesis and downregulating factors contributing to respiratory metabolism. The restriction of RuBP synthesis is probably related to the reduction in the synthesis of ATP [67]. Plants under drought stress exhibit a moderate increase in water-use efficiency, since a reduction in stomatal opening restricts transpiration more than the influx of CO_2_ [68]. This is due to a further increase in resistance to CO_2_ diffusion in the mesophyll, reducing the efficiency of carboxylation [67]. These results show that the treatments effectively induce severe stress, and demonstrate a reliable basis for further molecular analyses. The overproduction of ROS indicated a malfunction of the plasma membrane [69] and lipid peroxidation [67] during drought stress. It has also been reported that high ROS concentrations in plants are extremely toxic to lipids and result in oxidative stress. Therefore, these results indicate that the overproduction of ROS is the primary mechanism of water stress [70]. Compatible solutes such as carbohydrates, sugars alcohols (galactinol and mannitol), amino acids (proline), and amines (spermidine and glycine betaine) play important roles in adaptive mechanisms under drought stress. Osmoprotectants facilitate maintenance of cell turgor and cellular water potential under drought stress, as well as acting in membrane and macromolecule stabilization and ROS scavenging [71]. Various osmoregulatory substances, such as soluble sugars and soluble proteins, can increase the osmotic potential at the cellular level to prevent loss of moisture and enhance plants’ water-deficit stress resistance [72]. Moreover, complex mechanisms operate in plants to coordinate the interactions between carbon assimilation and nitrogen metabolism [73]. Carbon–nitrogen balance is a significant component in plants’ adaptation to water stress [74]. Proline synthesized via the glutamate or ornithine pathways is believed to act as a store of carbon and nitrogen, as well as affecting ROS scavenging. Accordingly, various studies have revealed that overexpression of either the glutamate or ornithine pathways in different plant species results in increased proline levels, which could contribute to enhanced stress tolerance [75]. Therefore, the gene co-expression network analysis provides an essential resource for mining novel and significant genes related to drought stress acclimation in soybeans. In particular, the hub genes and the genes involved in the largest clusters (i.e., turquoise, blue, and brown) are suggested to be the key players in soybeans’ drought stress response. Further downstream analysis studies will be essential in determining each of these hub genes’ contributions to drought stress tolerance in soybeans. 

To provide information regarding how genes are regulated in soybeans under drought stress conditions, we identified TFs as key molecules in the regulatory networks that play a central role in gene transcription and plants’ responses to drought stress. In the present study, there were 174 TFs in the DEGs belonging to 36 TF families (Table S4). Some of the major members of TF families—including orphan, MYB, AP2/ERF-ERF, and WRKY—were upregulated, while other major members of TF families—such as bHLH, C2C2-MYB-related, and C2C2-Dof—were found to be downregulated under stress conditions (Figure 6). The *C2H2* and *MYB* transcription factors are thought to be major transcriptional regulatory mechanisms in drought response. In this study, co-expression network analysis revealed that most TF transcripts connected with drought tolerance belong to the *C2H2* and *MYB* classes. The *C2C2* zinc finger class was also found to be related to secondary cell-wall biosynthesis in crops [76]. In recent years, numerous *MYB* transcription factors—mainly in the model species *Arabidopsis thaliana*, but also in some crops—have been characterized for their involvement in drought response. Multiple *MYB* TFs can be considered to be useful targets for biotechnological manipulation to improve drought resistance through overexpression or silencing. The expression of many *MYB* genes is regulated by drought. For example, in some crops and *A. thaliana*, 65% of *MYB* genes were expressed in seedlings and were differentially regulated under drought stress [77]. It has been shown that some of them play a specific role in response to water stress, such as the regulation of stomatal movement, the synthesis control of suberin and cuticular waxes, and the regulation of flower development. Moreover, some of these *MYB* genes play central roles in the control of plant-specific processes, including primary and secondary metabolism, cell fate and identity, development, control of cellular morphogenesis, response to abiotic and biotic stresses, and circadian rhythm [78]. 

Some MYB proteins are involved in responses to water stress through stomatal movements and the regulation of lateral root growth. Interestingly, the *MYB* TFs are positive regulators of lateral root growth through auxin signaling via interaction with the ABA receptor [79]. It has been indicated that lateral root growth is more sensitive to inhibition by ABA than that of wild-type seedlings in *myb77*-mutant plants. Exposure to auxin could reverse ABA-induced inhibition of lateral root growth in this mutant [80]. Generally, *MYB77* represents a key protein mediating crosstalk between ABA and auxin signaling in lateral root development in response to drought stress in soybeans.

Another important TF is C2H2-type zinc finger—a putative stress-associated gene, which is mainly expressed in the roots and stems, while subcellular localization analysis indicates that C2H2 is ubiquitously distributed in plant cells. Moreover, transgenic experiments have indicated that C2H2 plays a negative role in plants’ tolerance to water stress and might be involved in the ABA-dependent pathway during responses to drought stress. Therefore, given the expression patterns of marker genes related to stress or ABA, along with the effect of ABA on germination rates, it is clear that the function of C2H2 under water stress involves the ABA pathway but not the ROS pathway [81].

Although MYB, C2H2, and other TFs are useful candidate hub genes for improving drought tolerance in crops, the information acquired on MYB and C2H2 protein function so far has scarcely been applied to crop breeding. Taken together, these differentially expressed TFs might be involved in soybeans’ response to water-deficiency stress, and they could provide significant information for the study of drought tolerance in soybeans.

Analysis of the promoter regions of genomic sequences is often based on the detection of regions of the genome with transcription factor binding. Transcription factors are proteins that regulate gene transcription, and any change in their activity dynamically alters the transcriptome, causing metabolic and phenotypic changes in response to environmental stresses [82]. Therefore, investigating these regions and their transcription factors, along with downstream genes that are regulated by these proteins, is an attractive topic in the field of post-genomics and can provide new insights into critical metabolic pathways [83,84]. We performed a promoter analysis located upstream of the DEGs, and 11 conserved motifs with significant scores were identified (Table S5). Many cis-regulatory elements are related to water-deficient tolerance signaling pathways. The majority of the motifs found at the DEG promoters were highly matched to the MA128.1 and MA1278.1 motifs (Table 2 and Table S5), which are among the cis-acting elements of C2H2 zinc finger factors (DOF5.8). DOF TFs are zinc finger regulators and plant-specific transcription factors that play significant roles in vital processes and functions such as plant development, defense-regulatory networks, and responses to multiple biotic and abiotic stresses in plants [85]. The results of our study showed that *Arabidopsis* DOF5.8 is an upstream regulator of a gene encoding an NAC family member in response to abiotic stress [86]. Furthermore, a recent study has shown that the overexpression of this TF leads to a modification in the expression of many genes involved in drought/salt stress response, vascular tissue formation, photosynthetic carbon assimilation, dormancy and seed germination, secondary cell-wall deposition, and hormonal signaling during physiological processes [87]. This provides novel insights into the evolutionary and functional assays of the *Dof* gene family, which can aid in functional genomic studies of candidate *Dof* genes in order to genetically improve responses to drought stress in commercially important soybean cultivars.

PPI network analysis is a very useful tool to pinpoint associations between various genes—particularly those playing roles in a certain pathway [88]. It also delineates the putative interactions between TFs and their target genes. Interestingly, we observed a protein–protein interaction network consisting of key proteins such as *WRKY9, MYB140, FAD8, Ferredoxin-A*, and *GmCKX6-1*. In addition, the *WRKY9, MYB140, FAD8, Ferredoxin-A*, and *GmCKX6-1* genes had higher intramodular connectivity for the blue, brown, green, black, and turquoise modules, respectively. Modifying the expression of genes associated with the lipid metabolism pathway is one of the essential responses to stress conditions. Fatty acids are critical components of the cell membrane and are affected by environmental stresses. However, fatty acid desaturases (FADs) are a class of enzymes that mediate desaturation of fatty acids by introducing double bonds, helping the cell membrane to retain its function under stress by producing unsaturated fatty acids [89,90,91]. They play a significant role in modulating membrane fluidity in response to various abiotic stresses. Overexpression of two *FAD* genes (*FAD3* or *FAD8*) in tobacco improved its tolerance to drought and osmotic stresses [92]. In another study, silencing of *FADs* in tobacco plants reduced their levels of linolenic acid and resistance to drought and salinity stresses [89]. However, overexpression of *FADs* in soybeans resulted in increased levels of jasmonic acid and higher expression of *WRKY* as compared to mock-inoculated, vector-infected, and *FAD*-silenced soybean plants under drought and salinity stress conditions. Further investigation revealed that plants with overexpression of *FADs* showed higher chlorophyll content, photosystem II efficiency, relative water content, transpiration rate, stomatal conductance, and proline content, as well as a cooler canopy under drought and salinity stress conditions. However, *FAD*-silenced soybean plants were more sensitive to drought and salinity stresses [90].

Ferredoxins are known to increase water stress tolerance. Ferredoxins play significant roles in ROS scavenging, and their overexpression confers increased drought resistance in multiple systems [93,94]. Unique drought tolerance may involve producing additional ROS-scavenging ferredoxins. Moreover, ferredoxins are involved in photosynthesis in plants [95]. Thus, the main role of these proteins is to transfer electrons from photoreduced photosystem I (PSI) to ferredoxin NADP+ oxidoreductase (*FNR*), where NADPH is produced to aid in the assimilation of CO_2_.

The protein–protein interaction network analysis helped us to minimize the complexity in understanding the physical interactions between proteins in different stages of soybeans’ vegetative growth under drought stress. Along with transcriptomic analysis, the protein–protein interaction network analysis helped us to visualize and identify the key node proteins that affected water-deficit stress. Thus, the co-expression networks play a major role in identifying potential biomarkers of different abiotic stress conditions in plants by comparing different omics datasets pertaining to a specific functional context.

## 5. Conclusions

This study provides valuable information on soybeans in terms of differential and common host responses against drought stress. First, the meta-analysis and co-expression network analysis were used to select informative genes from high-dimensional gene expression data. Second, a cis-acting regulator analysis approach was used to identify motif promoters and TFs in a GCN. This study also shed some light on the mechanism of drought stress response in soybeans and discovered some key genes. Moreover, functional enrichment analysis of these key genes revealed their related intracellular functions under drought stress. This information revealed various molecular mechanisms, such as biosynthesis of secondary metabolites, photosynthesis, cytokinin dehydrogenase activity, and stress-specific roles of certain plant products that may be useful for the mitigation of drought stress in plants—particularly in soybeans. The key hub genes identified as candidate targets for bioengineering may provide new insights for developing drought-stress-resistant breeding and the genetic manipulation of crop plants by integrating and analyzing resistance traits. Defense-related pathways can be determined based on this, and they may simultaneously increase plants’ resistance to drought stresses and improve crop productivity. Further studies are required to elucidate the molecular mechanisms and validate the functions of responsive hub genes, TFs, and CREs that regulate plants’ responses to abiotic stresses.

## Figures and Tables

**Table 1:** Samples retrieved from GEO and ArrayExpress.

Accession	Type	Platform	Control	Treatment	Tissue	Released
E-GEOD-40627	Response to drought	GPL13674, Affymetrix	3	3	Leaf	10 November 2012
E-GEOD-65553	Response to drought	GPL13674, Affymetrix	9	9	Root	2 July 2015
E-GEOD-29663	Response to drought	GPL13674, Affymetrix	3	3	Leaf	2 April 2012
Total samples	30					

**Table 2:** Modules of all treatment studies.

Module Name	Number of Genes
Blue	441
Brown	258
Green	128
Red	99
Turquois	911
Yellow	220
Black	68
Pink	36

**Table 3:** The conserved cis-acting elements found in promoters of drought stress DEGs by MEME analysis.

Motif Name	Motif Logo	E-Value	Width	Best Match in JASPAR and PLACE	Significant GO Terms Identified by GOMO
Motif 1	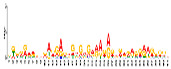	8.0 × 10^−100^	41	MA1403.1 (BBR/BBC)	MF: transcription factor activity CC: nucleus CC: plasma membrane BP: regulation of transcription, DNA-dependent BP: protein amino acid phosphorylation
Motif 2	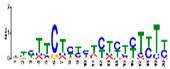	1.1 × 10^−44^	20	MA1268.1 (Dof)	MF: transcription factor activity CC: nucleus MF: protein serine/threonine kinase activity BP: protein amino acid phosphorylation BP: regulation of transcription, DNA-dependent
Motif 3	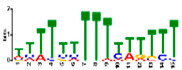	9.6 × 10^−27^	15	MA1268.1 (Dof)	MF: transcription factor activity BP: regulation of transcription CC: plasma membrane CC: nucleus BP: response to water deprivation
Motif 4	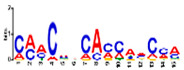	1.6 × 10^−17^	15	MA1890.1 (C2H2)	CC: chloroplast MF: transcription factor activity BP: photosynthetic electron transport in photosystem I
Motif 5	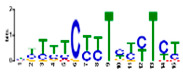	1.7 × 10^−5^	15	MA1403.1 (BBR/BBC)	MF: transcription factor activity CC: nucleus CC: plasma membrane BP: regulation of transcription, DNA-dependent MF: protein binding
Motif 6	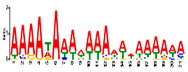	1.4 × 10^−10^	21	MA1281.1 (Dof)	MF: transcription factor activity CC: plasma membrane CC: nucleus BP: regulation of transcription BP: response to water deprivation
Motif 7	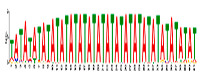	5.3 × 10^−5^	41	MA0386.1 (Fox)	MF: transcription factor activity CC: endomembrane system BP: regulation of transcription, DNA-dependent BP: oligopeptide transport MF: protein heterodimerization activity
Motif 8	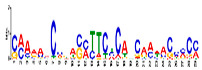	9.1 × 10^−1^	29	MA1354.1 (MYB-related)	CC: chloroplast
Motif 9	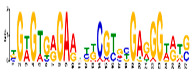	1.9 × 10^−3^	27	MA1892.1 (C2H2)	CC: chloroplast MF: transcription factor activity CC: nucleus BP: DNA replication initiation BP: developmental growth
Motif 10	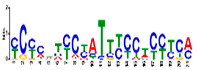	1.1 × 10^−4^	21	MA1723.1 (C2H2)	MF: transcription factor activity CC: nucleus BP: regulation of transcription, DNA-dependent CC: plasma membrane MF: protein binding
Motif 11	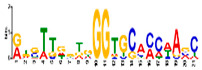	2.1 × 10^−4^	21	MA1766.1 (MYB)	

## Data Availability

Not applicable.

## Supplementary Materials

The following supporting information can be downloaded at: https://www.mdpi.com/article/10.3390/genes13101732/s1, Figure S1: Cross-validation analysis of ranked genes; Table S1: List of identified DEGs by meta-analysis of drought stress data, and their corresponding UniGene and transcript-stable IDs; Table S2: Gene Ontology annotation of modules found using the DAVID tool for drought stress data; Table S3: List of the top 30 hub genes in each module for drought stress data; Table S4: List of transcription factors enriched for drought stress DEGs; Table S5: List of known cis-acting elements among drought stress DEGs identified by a TomTom search against the JASPAR database; Table S6: Significant GO terms enriched by GOMO analysis for drought stress DEGs.
