# Supplementary material for: Integrative System Biology Analysis of Transcriptomic Responses to Drought Stress in Soybean (Glycine max L.)
Source: Genes (Basel). 2022 Sep 26;13(10):1732. doi: 10.3390/genes13101732 (PMC9602024; doi:10.3390/genes13101732)
Supplement: Supplementary file 1 [file genes-13-01732-s001.zip › genes-1833675-supplementary figure.pdf]

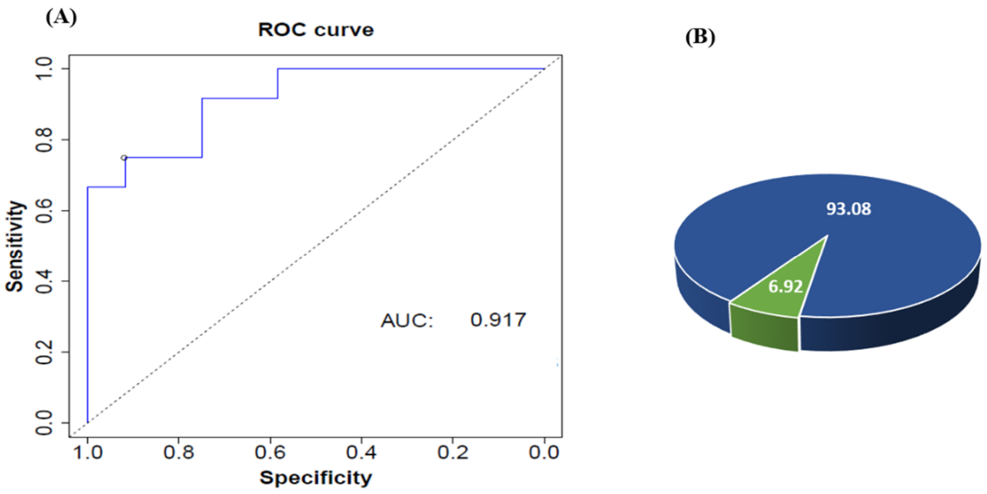

Figure S1. Cross-validation analysis of ranked genes (A) ROC curve for the hub genes. (B) The predictive accuracy (blue slice) and error (green slice) rate of LOOCV for hub genes.
